# The compendium of *Arabidopsis thaliana* GLYCINE-RICH RNA-BINDING PROTEIN 8 *in vivo* targets determined by iCLIP

**DOI:** 10.1038/s41597-025-05716-z

**Published:** 2025-08-06

**Authors:** Katja Meyer, Martin Lewinski, Tino Köster, Dorothee Staiger

**Affiliations:** RNA Biology and Molecular Biology, Faculty of Biology, Bielefeld University, Bielefeld, Germany

**Keywords:** Plant sciences, Molecular biology

## Abstract

RNA-binding proteins impact RNA processing including splicing, translation and RNA decay to dictate the fate of RNAs. Thus, insights into RNA-protein interactions in the cell provide insights into regulatory networks regulating gene expression at the posttranscriptional level. In higher plants, only few RNA-binding proteins have their *in vivo* targets and binding sites determined, mostly due to technical challenges posed by plant tissue. We previously adapted individual nucleotide-resolution crosslinking and immunoprecipitation that relies on UV-light irradiation to stabilize RNA-protein interactions in the cell to Arabidopsis plants. Here, we profiled the *in vivo* binding landscape of the glycine-rich RNA-binding protein *At*GRP8 by iCLIP. Transgenic plants expressing *At*GRP8 fused to GREEN FLUORESCENT PROTEIN under the native promoter were subjected to UV-crosslinking. RNA-protein complexes were immunoprecipitated and bound RNAs were determined by RNA-seq. A bioinformatics pipeline tailor-made for Arabidopsis identified target RNAs, and delineated binding sites and binding motifs for *At*GRP8. These data will aid in assembling a compendium of binding sites for plant RNA-binding proteins and contribute to unravel plant posttranscriptional networks.

## Background & Summary

The flow of genetic information from the genome to proteins is tightly regulated to ensure optimal plant performance. In particular, modulation of gene expression at the level of RNA has emerged as an important regulatory cue to integrate endogenous and external signals and adjust the plant transcriptome according to the needs. RNA-binding proteins (RBPs) play a key role in coordinating RNA processing and function during development and in response to abiotic and biotic stress factors^1–5^. In mammalian cells and non-plant model organisms, Individual-nucleotide resolution crosslinking and immunoprecipitation (iCLIP) is the gold standard to provide a genome-wide compendium of binding targets and binding sites with nucleotide precision^6,7^. UV light is applied to stabilize the native RNA-protein interactions in the cell. RNA-protein complexes are then immunoprecipitated from cell lysates and purified on denaturing gels. Bound proteins are digested, RNAs are isolated and subjected to reverse transcription to generate libraries for high throughput sequencing. As the remnants of the protein provide an obstacle to reverse transcriptase, the sequencing reads terminate at the crosslink site, thus revealing binding sites with single nucleotide resolution.

Knowledge about binding sites of RBPs in plants is scarce compared to binding sites in metazoan not least due to technical limitations due to the plant cell wall, the high RNase content of plant cells due to the vacuole, and the presence of UV absorbing pigments including the photosynthetic chlorophyll and the UV protective flavonoids^8,9^.

**Fig. 1:**
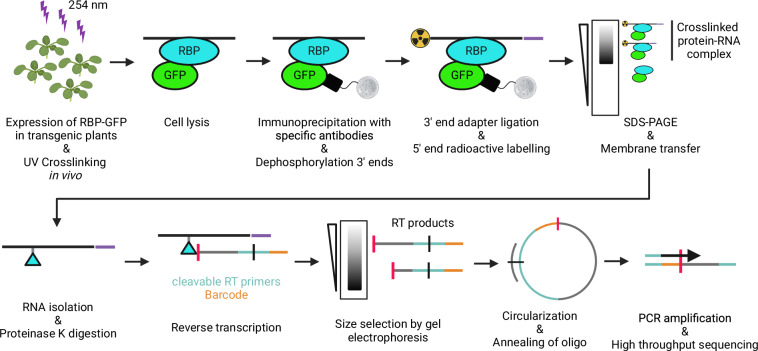
Experimental design of the study. An overview of the iCLIP protocol, adapted for transgenic Arabidopsis seedlings expressing an RNA-binding protein (RBP) fused to GREEN FLUORESCENT PROTEIN (GFP), is shown. See text for details.

We have previously performed the first iCLIP analysis in Arabidopsis plants to determine *in vivo* targets of a plant RBP, using the circadian clock regulated *At*GRP7 as a paradigm (Fig. 1)^9–11^.

Instrumental for a successful implementation of the iCLIP protocol originally developed for mammalian cells growing in monolayers to intact plant tissue was the use of antibodies that tolerate the harsh denaturing conditions necessary for efficient cell lysis such as single chain antibodies against common epitope tags raised in alpaca (nanobodies). Towards this end, we fused *At*GRP7 to GFP and expressed it under control of the native promoter and all regulatory sequences in a loss-of-function T-DNA line to maintain the endogenous expression pattern. Plants were grown in light-dark cycles and subjected to crosslinking 24 hours after transfer to continuous light. To ensure crosslinking in deeper layers of the tissue, we increased the UV dose to 500 mJ/cm^2^. For efficient cell lysis, we increased the concentration of SDS to 1%. Due to the large amounts of endogenous RNases, RNase inhibitors must be present during cell lysis and immunoprecipitation. To enable a controlled RNase treatment to fragment RNAs, we postponed the RNase treatment commonly performed in the cell lysate to the stage where RNA–protein complexes have been immunoprecipitated. After purification of the RNA-protein complexes and generation of cDNA libraries from the co-precipitated RNAs, we identified 525 target transcripts with *At*GRP7 binding sites.

More recently, a CLIP-seq analysis has been performed for *At*GRP7^12^. In contrast to iCLIP, CLIP-seq allows the identification of target transcripts but binding sites cannot be delineated precisely^12^.

*At*GRP7 constitutes a member of a limited cohort of glycine-rich RBPs in Arabidopsis^13,14^. The paralogous protein *At*GRP8 exhibits a remarkable 77.8% sequence identity at the protein level. This protein was identified owing to its modulation by the circadian clock and its response to cold, and it has consequently been designated as CCR1 (cold and circadian regulated 1)^15^. *At*GRP8 negatively autoregulates its expression and the expression of the paralogous *AtGRP7* while *At*GRP7 in turn negatively regulates *AtGRP8*. As such, these two proteins operate within an interlocked feedback mechanism that functions downstream of the circadian clock^16,17^.

Furthermore, *At*GRP8 was shown to be rapidly upregulated under oxidative stress, similar to *At*GRP7^18^. Recently, we have found that downregulation of *At*GRP8 in the *atgrp7-1* loss-of-function mutant enhances the delayed floral transition observed in *atgrp7-1*^19^. So far, direct *At*GRP8 *in vivo* target transcripts are elusive.

In pursuit of a comprehensive examination of the two paralogous proteins exhibiting overlapping functionalities, this report delineates the compendium of *At*GRP8 target transcripts and binding sites, using transgenic plants expressing *At*GRP8 fused to GFP under control of the endogenous promoter. With this, we identified 551 transcripts with *At*GRP8 binding sites. Above 70% of these are shared between *At*GRP7 and *At*GRP8, supporting the notion of common and specific functions of each of these paralogous proteins.

## Methods

### Plant material

The line *AtGRP8::AtGRP8:GFP* expresses an *At*GRP8-GFP fusion protein under control of 1.9 kb of the *AtGRP8* promoter and the native 5′UTR, 3′UTR and intron in the Col-2 background^20^. The *AtGRP7::AtGRP7 R*^*49*^*Q-GFP* line expressing an RNA binding mutant version of *At*GRP7 with Arg^49^ exchanged for Gln and the *AtGRP7::GFP* line expressing GFP only under control of the *At*GRP7 promoter have been described^21,22^.

Seeds were surface-sterilized and sown on half-strength MS (Murashige-Skoog) (Duchefa) plates^23^. Seedlings were grown in 12 h light/12 h dark cycles at 20 °C in Percival incubators (CLF laboratories) for 16 days and harvested 24 hours after transfer to continuous light (LL) in parallel to the seedlings expressing GFP only under control of the *AtGRP7* promoter and the seedlings expressing the RNA-binding dead variant *At*GRP7 R^49^Q described previously^10^. For each biological replicate, the material of three plates was pooled, yielding about 3 g of fresh weight.

### Individual-nucleotide resolution crosslinking and immunoprecipitation (iCLIP)

**Fig. 2:**
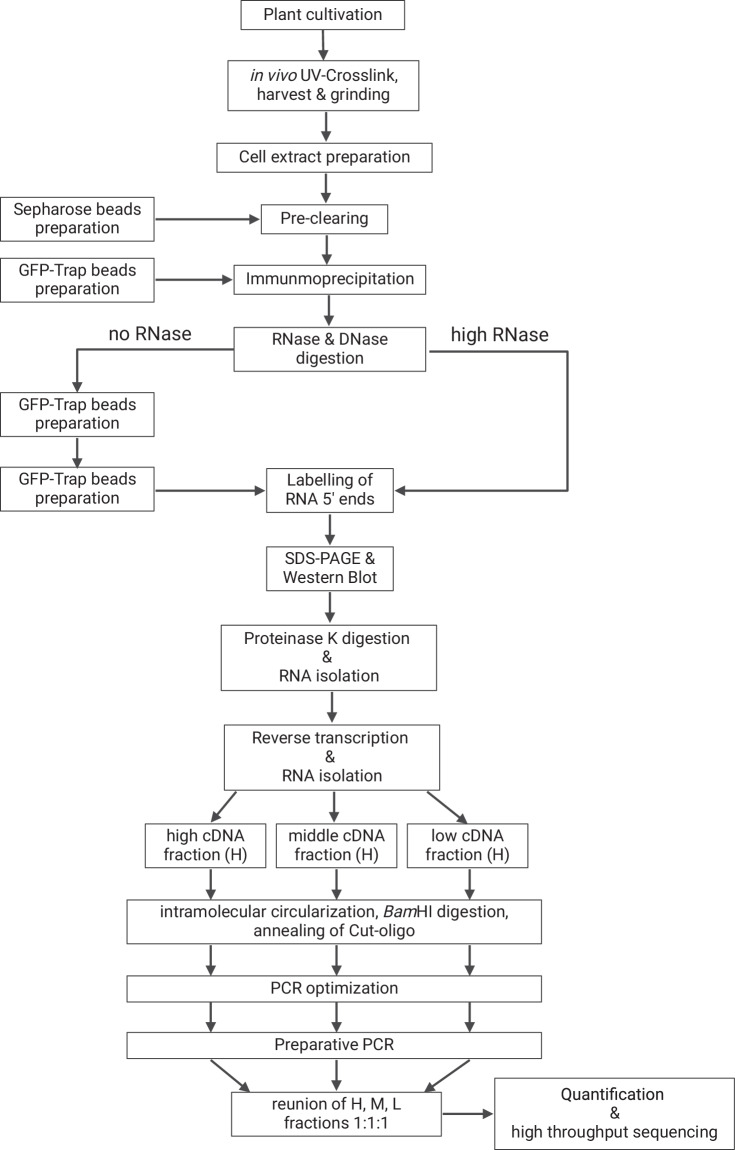
Workflow of the iCLIP procedure. Schematic overview of the wetlab workflow to determine RBP target transcripts and binding sites from *Arabidopssis thaliana* iCLIP data. See text for details.

iCLIP was performed as previously described^10^. The procedure is schematically shown in Fig. 1 and the workflow is presented in Fig. 2.

#### UV crosslinking

Transgenic *AtGRP8::AtGRP8:GFP s*eedlings grown on agar plates were subjected to irradiation with 254 nm UV light at a dose of 500 mJ/cm^2^ in a UVP CL-1000 UV crosslinker on ice. Seedlings expressing GFP only under control of the *AtGRP7* promoter and seedlings expressing the RNA-binding dead variant *At*GRP7 R^49^Q served as controls to take into consideration unspecific interactions of the GFP moiety^10^. Aerial tissue of the crosslinked material was harvested. The plant material was quick-frozen in liquid N_2_ and ground with mortar and pestle.

#### Immunoprecipitation

0.75 ml of cell lysis buffer (50 mM Tris-HCl, pH 7.5, 150 mM NaCl, 4 mM MgCl_2_, 0.25% Igepal CA-630, 1% SDS, 0.25% sodium deoxycholate, 5 mM DTT, Complete Protease Inhibitor (Roche), 100 U/mL RiboLock (Thermo Fisher), 1 mM phenylmethylsulfonylfluorid) was added to 0.5 g ground plant material. To reduce background binding, lysates were precleared with sepharose beads for 1 h at 4°C with constant rotation. After centrifugation, the supernatant was subjected to immunoprecipitation with GFP Trap beads (Proteintec) for 1 h at 4°C with constant rotation. The beads were then washed four times with 1 mL cooled RIP-washing buffer (50 mM Tris-HCl, pH 7.5, 500 mM NaCl, 4 mM MgCl_2_, 0.5% Igepal CA-630, 1% SDS, 0.5% sodium deoxycholate, sodium salt, 2 M Urea, 2 mM DTT, Complete Protease Inhibitor) and washed twice with 1 ml cold iCLIP wash buffer (20 mM Tris-HCl pH 7.4, 10 mM MgCl_2_, 0.2% Tween 20)^6^.

#### DNase and RNase treatment

On the beads, the precipitate was treated with 2 µl Turbo DNase for 10 min at 37°C (Thermo Fisher). For RNase digestion, 6.7 U RNase I (Thermo Fisher) were added.

#### Dephosphorylation and ligation of the 3′ linker

RNAs were dephosphorylated and the L3 linker (Table 1) was ligated to the 3′ends using RNA ligase (NEB).

**Table 1:** Linker and adapters used for generation of iCLIP libraries.

Oligonucleotides	Sequence 5′ → 3′
Cut_oligo	GTTCAGGATCCACGACGCTCTTCAAAA
GRP8clip1	NNACAANNNAGATCGGAAGAGCGTCGTGGATCCTGAACCGC
GRP8clip2	NNCCGGNNNAGATCGGAAGAGCGTCGTGGATCCTGAACCGC
GRP8clip3	NNGACCNNNAGATCGGAAGAGCGTCGTGGATCCTGAACCGC
P5Solexa	AATGATACGGCGACCACCGAGATCTACACTCTTTCCCTACACGACGCTCTTCCGATCT
P3Solexa	CAAGCAGAAGACGGCATACGAGATCGGTCTCGGCATTCCTGCTGAACCGCTCTTCCGATCT

#### Radioactive labeling

5′ termini were labeled using [^32^P]-ɤATP and polynucleotide kinase.

#### Denaturing gel electrophoresis and blot

The RNA–protein complexes were separated on a 4–12% NuPAGE Bis-Tris gel (Thermo Scientific), and electroblotted onto a nitrocellulose membrane. Upon autoradiography, the regions above the fusion protein were cut out.

#### RNA isolation and reverse transcription

The membrane regions were subjected to proteinase K treatment, leaving a polypeptide at the interaction site. Subsequently, RNA was isolated from the membrane using TriReagent and reverse transcribed using primers containing a cleavable adapter region and individual barcode sequences (Table 1).

#### Gel purification of cDNAs

After NaOH treatment, the cDNA was purified on a 6% urea-polyacrylamide gel and fragments in the size range of approx. 70–85 nucleotides, designated the low range, 85–120 nucleotides, designated the medium range, and 120–200 nucleotides, designated the high range were eluted from the gel and further processed.

#### Circularization and relinearization

The cDNAs were then circularized using CircLigase II (Epicentre) and the Cut-oligo was annealed to create a recognition site for *Bam*HI. Relineariztion via *Bam*HI digestion led to cDNAs with adapters at both ends.

#### PCR amplification and sequencing

Subsequently, the cDNAs were subjected to PCR amplification. After PCR optimization the three size fractions H, M, and L were pooled at a 1:1:1 ratio. Concentrations were determined with the Qubit dsDNA HS Assay Kit (Thermo Scientific), and 10 nM of the libraries were submitted to high throughput sequencing after multiplexing. Sequencing was carried out using an Illumina HiSeq 2500 at the Genomics Center of the Max-Planck-Institute for Developmental Biology, Tuebingen, with 50 nt single-end reads.

### Bioinformatics workflow

**Fig. 3:**
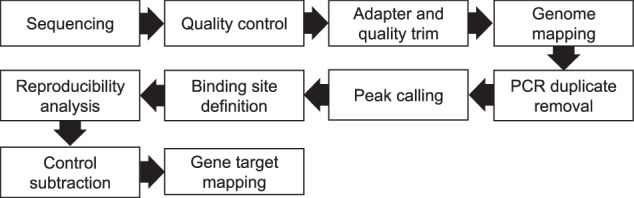
Bioinformatics workflow. Schematic workflow diagram of the bioinformatics steps beginning with the sequence analysis and quality control of the iCLIP reads until the final mapping to annotated genes. See text for details.

The bioinformatics workflow is schematically shown in Fig. 3.

#### Processing of iCLIP reads

Sequenced reads from all three replicates were analyzed in an initial quality assessment as well as after each processing step with *FastQC* (v0.11.5) with standard parameters. Adapter sequences at the 3′ ends were trimmed using *Cutadapt* (v1.16) with parameters –a AGATCGGAAGAGCGGTTCAGCAGGAATGCCGAGACCGATCTCGTATGCCGTCTTCTGCTTG -j 3 to specify the reverse complemented P3Solexa adapter (Table 1) and three multiprocessing threads. Demultiplexing was performed with *Flexbar* v3.4.0^24,25^, using the –bk parameter to retain barcode information for subsequent steps. Reads shorter than 24 nucleotides were removed (-m 24) from further analysis as they are considered too short for unique genomic mapping. In the same process barcodes were concatenated to the end of the read ID field for later PCR duplicate removal. The average read quality and lengths have been computed using *bioawk* (https://github.com/lh3/bioawk). High-quality reads were mapped to the TAIR10 genome using *STAR* (v2.6.0a), allowing up to two mismatches. To enhance alignment hits, soft-clipping at the 5′ end was disabled, using the parameter --alignEndsType Extend5pOfRead1. The size of introns for alignments was limited between 11 and 280000 nucleotides (--alignIntronMin 11 –alignIntronMax 28000) and only uniquely mapped reads were reported (--outFilterMultimapNmax 1). PCR duplicates, artifacts resulting from library amplification that share identical unique molecular identifiers (UMIs), were removed by grouping reads based on their mapping start positions and eliminating reads with identical start positions and random barcode sequences. An overview of read counts after each processing step is listed in Table 2.

**Table 2:** Read statistics of *At*GRP8-GFP iCLIP with three replicates after each processing step.

Reads	Replicate 1	Replicate 2	Replicate 3
Demultiplexed and quality trimmed	3,157,443	3,281,257	3,280,019
Successfully mapped	2,940,751	3,066,601	3,109,179
Mapped uniquely	1,338,793	1,727,565	1,219,287
Mapped to multiple locations	1,601,958	1,339,036	1,889,892
Mapped %	93.14%	93.46%	94.79%
Mapped % (unique)	42.40%	52.65%	37.17%
After PCR duplicate removal (unique)	547,295	844,177	345,130

#### Peak calling

In iCLIP libraries the most valuable information originates from the direct interaction between the protein and RNA (crosslink) and not the mapped read sequence. Therefore from mapped reads only the crosslink site (read start −1) is utilized for the downstream analysis and peak positions are reported in regard to the crosslink sites. Peak calling was conducted with *PureCLIP*^26^ (v1.0.4) in narrow-peak mode (-bc 0), restricted to uniquely mapped reads (Table S1). For adjacent called peaks (one nucleotide resolution), only the peak with the highest PureCLIP score was retained for further processing to minimize redundancy. Binding sites were defined as peak positions (single nucleotide resolution) extended by 4 nucleotides on both directions, which was computed with *Bedtools* (v2.27.1). Binding sites containing a single crosslink position within the 9-nucleotide window were excluded from further analysis as they are considered mapping artifacts (Table S2). Reproducibility across replicates was estimated by overlapping binding sites with crosslink coordinates from each sequenced replicate. A 30% quantile threshold was applied to filter binding sites with low support coverage from each individual replicate. Subsequently, binding sites were classified as reproducible if they had crosslink counts above the defined 30% threshold and were present in two out of three replicates. The final set of *At*GRP8 reproducible binding sites was created by subtracting overlapping sites from the *At*GRP7-R^49^Q and GFP-only^10^ control samples (Table S3). Each remaining binding site was assigned to a transcript region (5´ untranslated region (UTR), 3´ UTR, intron, coding region or non-coding genes) by considering representative gene models from the Araport11 protein-coding and non-coding gene annotation (Table S4).

### Determination of binding motif

The binding distribution of *At*GRP8 on protein coding target transcripts was computed using Bedtools and the Araport11 gene annotation and compared to the total genomic length of the features (Fig. 3). For *de novo* motif discovery reproducible binding sites of *At*GRP8 (Table S4) were extended by 30 nucleotides in both directions and genomic sequences extracted in strand specific mode (-s) to FASTA format using *Bedtools* (v2.27.1) using the TAIR10 genome in FASTA format. The motif discovery tool *STREME* (v5.5.7) was then applied on these FASTA sequences to determine significantly enriched sequence motifs around the binding site centers (Fig. 3).

## Data Records

The demultiplexed iCLIP-seq reads for *At*GRP8-GFP have been submitted to the Sequence read Archive (SRA) at the National Center for Biotechnology Information (NCBI) under the accessions SRR32329121^27^, SRR32329122^28^, and SRR32329123^29^.

### External data files

1. Araport11 gene annotation BED files: protein-coding and non-coding gene locations on the TAIR10 genome^30^.

The processed data files for called peak coordinates, binding sites, and reproducible binding sites are provided as supplemental Excel sheets (Supplementary Table S1) in BED (browser extensible data) column format. Files with raw iCLIP signals are provided for each individual replicate and in merged form as in BEDGRAPH files via figshare^31^. The table with determined *At*GRP8 iCLIP target transcripts derived from reproducible binding site coordinates is provided in Table S4.

## Technical Validation

### Biological repetition

**Fig. 4:**
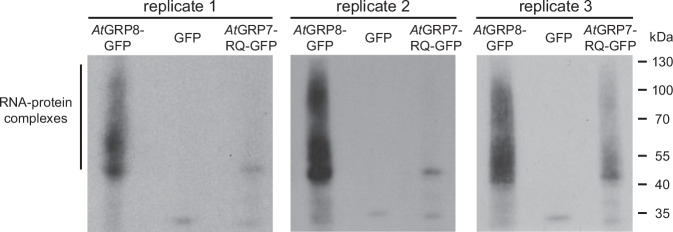
Control of RNA-protein complexes precipitated from plants expressing *At*GRP8-GFP. Autoradiogram of the RNA-protein complexes precipitated from plants expressing *At*GRP8-GFP and from the control plants expressing the RNA-binding dead *At*GRP7-R^49^Q-GFP, or GFP alone after radioactive labeling, separation on a 4–12% NuPAGE Bis-Tris gel and transfer onto a nitrocellulose membrane. The *At*GRP8-GFP samples show a broad smear of the bound RNAs in all three replicates, whereas *At*GRP7-R^49^Q-GFP and the GFP control only show a weak radioactive signal. The region cut out from the membrane and used to generate iCLIP libraries is indicated by the vertical bar on the left side. The positions of the molecular weight standards are indicated. Shown are the results for three independent biological replicates.

For this study, three biological replicates were independently processed (Fig. 4).

### Data quality assessment

The dataset consists of three replicates of *Arabidopsis thaliana* iCLIP reads with a length of 50 nucleotides. The reads were sequenced on an Illumina HighSeq 2500 platform in single end sequencing. Due to the experimental procedure, the interaction site (crosslink) between the fusion protein *At*GRP8-GFP and its target RNA is not part of the reads as the reverse transcription terminates right before the crosslink site (Fig. 1). Therefore, the mapped coordinates of iCLIP reads are used as an anchor point to detect transcriptome-wide protein-RNA interactions. Each replicate consists of over 3 million reads (Table 2) in high quality (Ø Q40), which is above the threshold what is considered a good quality iCLIP library^32^. After peak calling and binding site definition (details see methods section) the reproducibility is determined by backtracking the number of reads (crosslinks) to the binding sites (Fig. 3) and therefore remove binding sites with a low reproducibility. The amount of crosslinks overlapping the defined binding sites is expected to form a log-normal distribution is to be expected^32^ and can vary for each replicate depending on the overlapping crosslink signal and number of reads in the library.

**Fig. 5:**
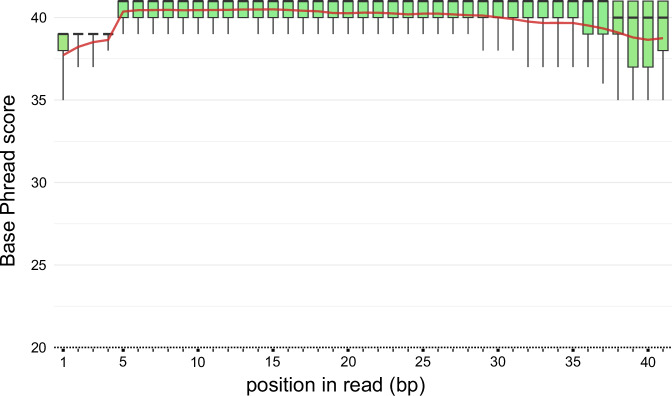
Per Base Sequence Quality of *At*GRP8-GFP library reads after quality processing. The boxplots indicate the distribution of read quality in Phred scores for each sequenced position, reported by FastQC. The scores are plotted along the y-axis, and the base position along the x-axis. The regions above 28 indicate high quality scores (error < 0.2%). Boxes display the upper and lower quartile, whiskers indicate the upper and lower 10% percentile of quality scores. The red line shows the average values at each base position.

**Fig. 6:**
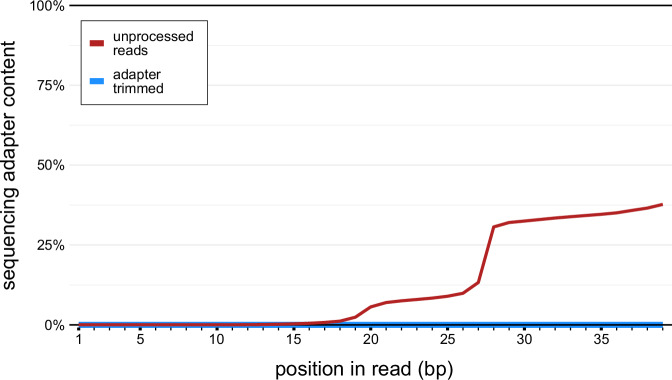
Adapter content of *At*GRP8-GFP library reads before and after adapter removal. The line chart presents the identified adapter positions per base in the *At*GRP8 iCLIP reads before and after 3’ adapter trimming, as reported by FastQC. The red line indicates the adapter presence before trimming, showing a significant increase toward the end of the read. The blue line represents the adapter content after trimming, demonstrating a marked reduction and near elimination of adapter contamination across all base positions.

The quality of the iCLIP reads was assessed using FastQC (Fig. 5). We confirmed using *bioawk* that the base quality was on average Q40 (±1.42) and the read length after adapter removal was 30.8 (±10) bp. In total 1.7 million reads were left after quality processing, unique mapping and PCR duplicate removal (Table 2). Figure 6 shows evidence of the successful adapter removal.

**Fig. 7:**
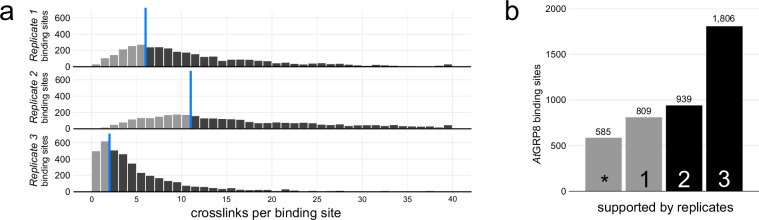
Reproducibility of *At*GRP8-GFP binding sites. (**a**) Crosslink distribution across binding sites, with light grey bars representing sites below the 30% quantile threshold (blue line). (**b**) Bar chart of binding sites surpassing the threshold across n (1–3) replicates. The asterisk (*) highlights binding sites below the 30% quantile threshold.

To determine the correlation between replicates we have calculated the number of crosslinks coming from each of the individual replicates (Fig. 7a). The crosslink-distribution of the defined binding sites forms a log-normal distribution. We have set a 30% quantile threshold to determine the minimal threshold of crosslinks to define reproducible binding sites (R1: 7, R2: 12, R3: 3). Of these 4.139 binding sites a major part coincides with signal from at least two out of three replicates (66.3%) and also a significant part of binding sites in all of the three replicates (43.6%) after applying the filtering thresholds (Fig. 7b).

**Fig. 8:**
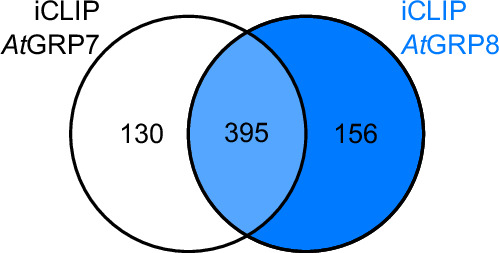
Venn Diagram of *At*GRP8 and *At*GRP7 iCLIP targets. Venn diagram of target transcripts of the *Arabidopsis thaliana* RNA binding proteins *At*GRP8 (blue, this study) and *At*GRP7 (white^10^) determined by iCLIP.

In order to ascertain the extent of overlap between the binding targets of *At*GRP8 identified in this study and those of its closely related paralog *At*GRP7 delineated in prior research^10^, we intersected both data sets. At the level of target transcripts, which possess at least one reproducible binding site, our findings indicate that above 70% of the *At*GRP8 target transcripts were also bound by *At*GRP7 (Fig. 8).

**Fig. 9:**
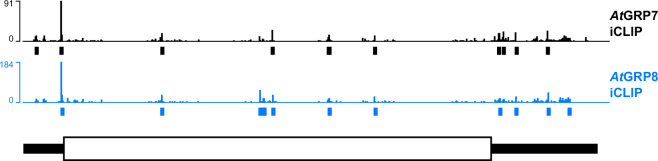
Crosslink and binding site tracks of *At*GRP7 and *At*GRP8 iCLIP on the *LCHB1.1* transcript. Genomic tracks of *At*GRP8-GFP and *At*GRP7-GFP crosslinks and reproducible binding sites determined on the target transcript encoding *LIGHT HARVESTING CHLOROPHYLL A/B-BINDING PROTEIN 1.1* (*LHCB1.1*). The data tracks (bars) show cross-link positions and read counts in single-nucleotide resolution resulting from the bioinformatics workflow. Boxes below the cross-link track mark the position of reproducible binding sites. Data from *At*GRP7 iCLIP^35^ are colored black and data from *At*GRP8 in blue, respectively. The corresponding gene model is depicted in the bottom track in block format. Thick white black bars represent the coding region, thin black bars are the 5′ and 3′ UTRs.

One of the common target genes identified by iCLIP was *LIGHT HARVESTING CHLOROPHYLL A/B-BINDING PROTEIN 1*.1 (*LHCB1.1*). Given that *At*GRP7 was previously shown to regulate *LHCB1.1* expression, we undertook a thorough comparative analysis of the crosslinking distribution and the positional arrangement of binding sites on this gene. A direct comparison of the data revealed that both, the raw crosslinking signals and the reproducible binding sites exhibit a congruent binding pattern of the paralogous proteins, alongside a pronounced positional correlation on the target RNAs (Fig. 9).

**Fig. 10:**
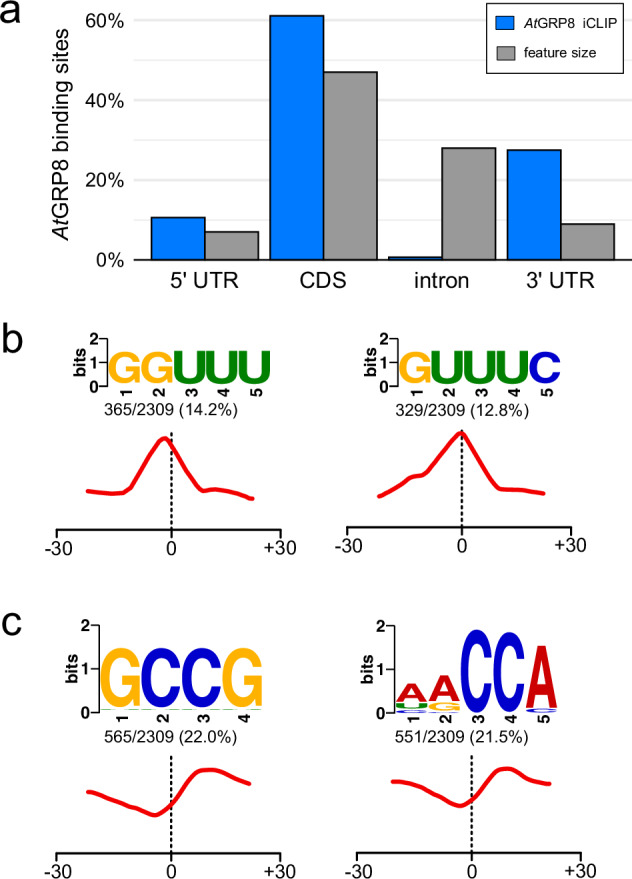
*At*GRP8 binding motif. (**a**) Distribution of *At*GRP8 binding sites within protein coding transcripts and percentage of reproducible binding sites in different transcript regions (blue bars) compared to the percentage of cumulative length of the indicated region (light grey bars) based on representative gene models from Araport11. (**b**) Significantly enriched sequence motifs overlapping *At*GRP8 binding sites and (**c**) motifs enriched downstream of *At*GRP8 binding sites computed with STREME. The red line-graph below the sequence motifs describes the density of each motif in relation to binding site centers (dotted line).

A fundamental component of iCLIP data is the elucidation of binding patterns throughout the entire transcriptome, which can only be achieved by acquiring high-resolution data on binding sites. These binding patterns subsequently enable the exploration of the functional roles of the protein^33^. Furthermore, a comparative analysis with other data sets provides the means to identify whether the proteins work cooperatively or antagonistic^34^. A positional analysis of binding sites revealed that *At*GRP8 binding sites are predominantly located in untranslated regions (UTRs) (Fig. 10a), a finding that aligns with observations made for *At*GRP7. The core motif enriched at the centre of *At*GRP8 binding sites also parallels the motif identified for *At*GRP7 (Fig. 10b). In addition to the core motif, we were able to identify motifs that do not correspond to the motifs of either *At*GRP7 or *At*GRP8 and are located around 10 nucleotides downstream of the identified binding sites (Fig. 10c). One may speculate that these could be potential binding sites for RBPs that have not been characterized yet.

**Fig. 11:**
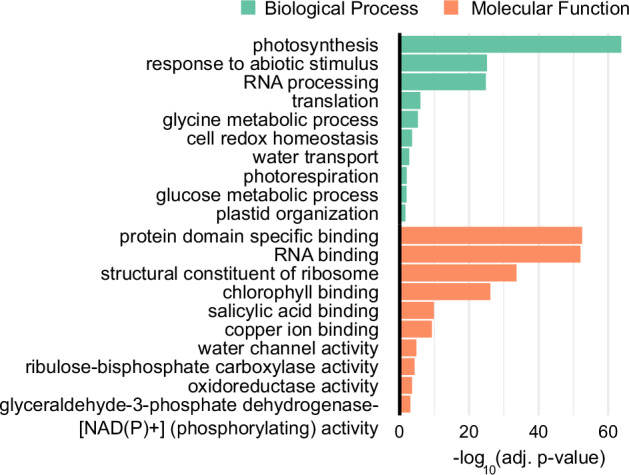
Gene Ontology analysis of *At*GRP8 iCLIP targets. Bar plots illustrating significantly enriched Gene Ontology (GO) terms in the categories *Biological Process* (green) and *Molecular Function* (orange) for *At*GRP8-GFP iCLIP target transcripts determined using g:Profiler^36^. The x-axis represents -log_10_ transformed adjusted p-values for each GO term, indicating the significance of enrichment.

GO term analyses of the direct target transcripts of *At*GRP8 point to the involvement in distinct biological processes including photosynthesis, response to abiotic stress and RNA processing (Fig. 11). This is well reflected by the well-described control of *At*GRP8 by abiotic stimuli and the function of *At*GRP8 in pre-mRNA splicing^15,22^.

Overall, the quality of the *At*GRP8 iCLIP libraries is reflected in their large read counts, high base-calling scores, and mapping rates. Moreover, their reproducibility and close similarity to the paralog *At*GRP7 further reinforce their robustness and provides insights into the binding landscape of an additional RNA-binding protein in plants.

## Usage Notes

TAIR10 genome FASTA contains genomic sequences of all chromosomes of the current *Arabidopsis thaliana* genome (https://www.ncbi.nlm.nih.gov/datasets/genome/GCF_000001735.4/).

## Supplementary information

Supplementary Table S1

## Data Availability

The analysis of iCLIP reads was performed according to our previously reported bioinformatics pipeline^35^. The bioinformatics workflow, from quality control to the identification of reproducible binding sites, is described in detail in the online repository at https://github.com/malewins/iCLIP-AtGRP7-pipeline.
